# COVID and early intervention: the impact of COVID-19 on referrals to an early intervention service

**DOI:** 10.1192/bjo.2021.329

**Published:** 2021-06-18

**Authors:** Adam Whyte, Alastair Reid

**Affiliations:** Oxford Health NHS Foundation Trust

## Abstract

**Aims:**

COVID-19 has a demonstratable impact on the population's mental health and is associated with an increased incidence of psychiatric disorders, including patients experiencing psychotic presentations. The aim of this study was to explore whether referral rates within a county-wide Early Intervention (EI) service changed in response to the COVID-19 pandemic. The EI service provides NICE approved treatments and support for patients experiencing a First Episode Psychosis (FEP).

**Method:**

Data were collected from all referrals to the EI service between March–December 2019 and March–December 2020. Clinical notes were reviewed to ascertain whether the referred patient was assessed and if they were subsequently accepted on to the team's caseload.

**Result:**

During the March–December 2019 period 147 referrals were made to the EI service, with 66 patients being accepted for treatment by the service (44.9% of referrals). In March–December 2020, 127 referrals were made, a 13.6% reduction compared to the same period in 2019, however 70 referrals were accepted (55.1% of referrals).

Whilst the overall referrals declined during the COVID-19 period, there were notable increases in both April and August 2020, by 25.0% and 70.0% respectively.

**Conclusion:**

Although overall referrals to the EI service reduced during the COVID-19 pandemic compared similarly to the previous year, there was a noteworthy increase in the proportion of patients accepted onto the team's caseload.

Potential explanations for this finding include the possibility of an increased incidence of first episode psychosis during this period, or that restrictions in accessing primary care and secondary mental health services during the COVID-19 pandemic reduced the number of patients being referred whose symptoms were not representative of First Episode Psychosis (FEP).

This study highlights that mental health services, such as EI teams, have experienced a persistent level of need over the past year and that ongoing investment in psychiatric services is warranted to meet this sustained requirement for support and interventions.

